# The Antidepressant Effect of Deoiled Sunflower Seeds on Chronic Unpredictable Mild Stress in Mice Through Regulation of Microbiota–Gut–Brain Axis

**DOI:** 10.3389/fnut.2022.908297

**Published:** 2022-07-01

**Authors:** Xiaomeng Lu, Ce Qi, Jie Zheng, Mei Sun, Long Jin, Jin Sun

**Affiliations:** ^1^Institute of Nutrition and Health, Qingdao University, Qingdao, China; ^2^National R&D Center for Nuts Processing Technology, Qiaqia Food Co., Ltd., Hefei, China

**Keywords:** deoiled and dechlorogenic acid sunflower seeds, antidepressant, chronic unpredictable mild stress, oxidative stress, intestinal mucosal barrier, gut microbiota

## Abstract

**Objectives:**

Sunflower seeds provide tryptophan-rich proteins with the potential to protect against depression. Tryptophan is a precursor of serotonin and a substrate for the production of indole derivatives by gut microbiota. This study aimed to investigate the association between the depression-alleviating effects of deoiled and dechlorogenic sunflower seeds (DSFS) and regulation of gut microbiota.

**Materials and Methods:**

Male C57BL/6J mice were fed a diet comprising a source of soy protein (normal and model control), DSFS or whey protein concentrate (positive control) for 7 weeks, and chronic stress-induced depression was induced.

**Results:**

Feeding the DSFS diet prevented depression-like behaviors, intestinal barrier damage, elevated plasma corticosterone, and reduced hippocampal serotonin levels in mice. Meanwhile, Feeding the DSFS diet significantly altered the gut microbiota structure, characterized by elevated relative abundances of *Ileibacterium valens*, *Ruminococcus flavefaciens*, *Clostridium scindens*, and *Olsenella massiliensis*, which were inversely associated with depressive behaviors and markers of mucosal barrier damage. DSFS also altered the gut metabolite profile, prevented depression-induced gut L-tryptophan depletion, and upregulated its metabolite indoleacetaldehyde.

**Conclusion:**

Feeding the DSFS diet prevented depression in mice by remodeling the gut microbiota and bacterial tryptophan metabolism.

## Introduction

Depression is a complex psychiatric and emotional disorder, that generally results in a range of symptoms, such as low mood, anhedonia, lack of attention, sleep disorders, loss of interest, and a low-level of general vigor (1). Depression affects nearly 350 million people worldwide and contributes to a heavy economic burden and tremendous pressure on families and society (2). Although many types of antidepressant drugs have been developed to treat depression, most have limitations and adverse side effects (3).

Recently, the relationship between a high tryptophan (Trp) diet or Trp metabolism and depression has been widely studied. A single dose of Trp can produce a dose-dependent antidepressant effect, which mainly depends on the efficiency of Trp conversion into 5-hydroxytryptamine (5-HT) (4). Dietary interventions rich in Trp have also demonstrated good antidepressant/anxiolytic effects. Certain natural products such as deoiled gourd seeds (Trp: 22 mg/g protein) in combination with glucose can improve individual social anxiety and achieve clinical effects similar to those of medical-grade Trp (5). Compared with other food protein sources, whey protein or whey-derived protein contains a relatively high concentration of Trp, which is superior in relieving stress (6). A whey protein diet has been reported to enhance 5-HT synthesis, reverse behavioral disorders in stressed mice, and reduce the cortisol response and anxiety state of stressed subjects (7, 8). Sunflower seeds are a high-Trp/protein food. The Trp content is 1.75 g/100 g protein, second only to milk and sesame (9). Our study confirmed that deoiled sunflower seeds high in Trp levels can improve the utilization of Trp and promote the synthesis of 5-HT in the brain, thereby improving behavioral disorders and depression in stressed mice (10). However, the specific mechanisms involved require further investigation.

Tryptophan in the gastrointestinal tract is metabolized by the gut microbiota into indole derivatives, such as indole-3-acetic acid and indole-3-propionic acid (11). These metabolites cross the blood-brain barrier and activate aryl hydrocarbon receptors in astrocytes and microglia (12). The activation of aryl hydrocarbon receptors (AhR) can inhibit the pro-inflammatory transcription factor nuclear factor-κB pathway, which prevents the production of pro-inflammatory cytokines and contributes to maintaining integrity of the intestinal mucosal barrier (13). The severity of intestinal mucosal barrier injury has been reported to be positively correlated with the degree of depression (14).

Furthermore, the gut microbiota affects the modulation of brain function via the microbiota–gut–brain axis. Many researchers have suggested that gut microbiota disorders are strongly highly related to the occurrence of depression (15). Numerous animal models of depression have demonstrated that the gut microbiota is disordered (16). The gut microbiota can alter the behavior and function of the central nervous system by regulating gut barrier integrity and the development of inflammation, thereby stimulating the immune system and enhancing the secretion of neurotransmitters (17). Crucially, increased gut permeability and perturbed gut microbiota are believed to be related to the development of many neuropsychiatric disorders (18). However, the mechanisms underlying gut microbiota disorders that promote the development of depression and their effects on the host, are not fully understood.

An imbalance in redox homeostasis is the main cause of depression (19). Chronic mild stress induces oxidant production and disturbs the activities of superoxide dismutase and catalase, which may cause oxidative stress, thereby contributing to the development of depression (20). Additionally, increased oxidative stress activates pro-inflammatory signaling pathways that can lead to depression (21). Inflammatory cells produce reactive oxygen species (ROS), which in turn promote the expression of pro-inflammatory genes (19). Furthermore, the antidepressant response involves in changes in oxidative and inflammatory markers. High levels of ROS and inflammatory biomarkers in patients with depression, along with activated stress kinases, promote further oxidative stress and neuroinflammation, leading to cell death, all of which might contribute to depression (22).

Hence, we hypothesized that deoiled and dechlorogenic sunflower seeds (DSFS) may prevent depression by regulating gut microbiota metabolism and improving the intestinal mucosal barrier. This study aimed to estimate the effect of DSFS (Trp: 2.35/100 g protein) on chronic unpredictable mild stress (CUMS)-induced depression-like states in C57BL/6J mice. We explored the effects of DSFS on the intestinal mucosal barrier, oxidative stress, gut microbiota, and metabolic profiles.

## Materials and Methods

### Preparation of DSFS

Sunflower seeds were provided by Qiaqia Food Co. Ltd. (Hefei, China). Whey protein concentrate (WPC) and soy protein were purchased from Qinuo Food Co. Ltd. (Zhengzhou, China) and Jiangsu Fu Shing Tak Biological Engineering Co. Ltd. (Nanjing, China), respectively. Sunflower seeds were deoiled by cold pressing to prevent oxidation and loss of Trp. Sunflower seeds contain many polyphenol compounds, especially chlorogenic acid, which accounts for up to 70% of total polyphenols. Chlorogenic acid readily interacts with polar groups, resulting in difficult absorption of the protein (23). Ultrasonic-assisted ethanol extraction was used to remove chlorogenic acid to obtain DSFS. Amino acid levels of the three proteins were measured according to the method described by Ren et al. (24). Supplementary Table 1 presents the amino acid compositions identified.

### Animals

Forty male C57BL/6J mice (6-weeks old, weighing 18–21 g) were kept in the Experimental Animal Center of Jiangnan University under standard laboratory conditions of a natural 12 h light and dark cycle, at a constant temperature (23 ± 2°C), and under constant humidity (60 ± 5%). The mice were free to eat and drink *ad libitum*. All experimental procedures were approved by Jiangnan University Animal Care and Use Ethics Committee (JN. No20181230c0480710 [281]) and were performed according to the National Guidelines for Ethical Review of Experimental Animal Welfare.

**FIGURE 1:**
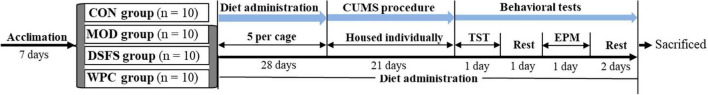
Schedule of the animal experiment. CUMS, chronic unpredictable mild stress; TST, tail suspension test; EPM, elevated plus maze test. CON, normal control group; MOD, model control group; DSFS, deoiled and dechlorogenic acid sunflower seeds group; WPC, whey protein group (*n* = 10 per group).

After 7 days of acclimatization, mice were randomly assigned to four groups (*n* = 10 per group): CON, which was not subjected to any stress and received a soy protein diet; the other three groups were subjected to CUMS induction and received a soy protein diet (MOD), a DSFS diet and a WPC diet. Diets were prepared for AIN-93M diet formulation (Supplementary Table 2; 25). After 28 days of dietary intervention, the mice were maintained separately and subjected to CUMS for 21 days. Figure 1 presents the detailed experimental procedures.

### Chronic Unpredictable Mild Stress

The CUMS model, a well-validated rodent model of depression, is based on a series of unpredictable mild stressors to simulate the social stress experienced by humans in daily life (26). Long-term exposure to stress can lead to anhedonia, changes in behavioral activities, and deterioration in appearance, which eventually evolve into various mental illnesses or emotional disorders such as depression and anxiety (27). In summary, mice were exposed to any two unpredictable stressors each day (28): 2 h of approximately 45° tilted cages, 3 min of tail pinching, 4 h of bedding removal, 3 h of cage breeding (five per cage), 12 h of food and water deprivation, 3 h of exposure to rat feces, 3 h of bedding removal and addition of water to a depth of 0.25 inches, and a reversal of the diurnal cycle (12/12 h light/dark).

### Behavioral Tests

#### Tail Suspension Test

The tail suspension test (TST) was performed to measure the total immobility time (28). Mice were hung 20 cm above the floor by taping the tail to a stabilizer bar for 6 min, and the immobility time was recorded over the last 4 min. Mice were considered immobile only when they hung vertically and motionlessly.

#### Elevated Plus Maze

The elevated plus maze (EPM) test was performed to assess anxiety. The instrument contained two open and two closed arms (50 cm × 5 cm). They were joined together by a central area (5 cm × 5 cm) and placed 45 cm above the floor. The closed arm was enclosed by an opaque 30 cm wall. The mice were placed in the center and allowed to explore freely for 5 min.

#### Open Field Test

The open field test (OFT) was used to measures depression/anxiety-like behavior. In the dark room, mice were housed in a topless box (40 cm by 40 cm by 30 cm). A camera is mounted above the box, and a maze video tracking system is used to track and record the mice activity. In the system, the bottom part of the box is divided into 16 small square grids (10 cm × 10 cm). The four-square grids of the central region are defined as the central region. The mice were placed in the central area of the device and allowed to explore freely for 30 min.

### Sample Collection

After the behavioral tests, mice were sacrificed using the cervical dislocation method. Blood was collected and centrifuged (4,000 × *g*, 10 min, 4°C). The liver was rapidly homogenized to determine ROS levels. The colon was collected for RNA extraction, and the content was frozen for subsequent *16S* ribosomal gene sequencing and metabolic profiling.

### Biochemical Analysis

Plasma corticosterone, endotoxin (ET), diamine oxidase (DAO) and 5-HT levels were determined using enzyme-linked immunosorbent assay (ELISA) kits, according to the manufacturer’s instructions (Jiangsu Meimian Industrial Co. Ltd., Yancheng, China).

### Measurement of Oxidative Stress Levels

Liver superoxide dismutase (SOD), glutathione peroxidase (GSH-Px), catalase (CAT), and malondialdehyde (MDA) levels were quantified using ELISA kits (Nanjing Jiancheng Bioengineering Institute, Nanjing, China). ROS levels were measured as described by Kobayashi et al. (29).

### Histopathological Analysis of Ileum Tissue

The ileum tissue was first fixed with paraformaldehyde, then dehydrated, embedded, sectioned, and stained with hematoxylin and eosin (H&E).

### Real Time qPCR Analysis

Total RNA was extracted using Trizol (Biomiga, Shanghai, China), purity was determined by measuring the A260/A280 ratio, and the quality was assessed by agarose gel electrophoresis. Total RNA was then reverse-transcribed using a Moloney Murine Leukemia Virus Reverse Transcriptase kit (MultiScribe Reverse Transcriptase; Applied Biosystems). mRNA expression was quantified by qPCR using the following thermocycling conditions: 1 cycle at 95°C for 5 min; 40 cycles at 95°C for 20 s, 60°C for 30 s, and 72°C for 20 s, and one cycle at 72°C for 2 min (Monad Biotech Co., Ltd., Suzhou, China). PCR primer sequences are listed in Supplementary Table 3. Gene expression was calculated as a relative value using β-actin as a reference, according to the comparative threshold cycle (Ct) method (30).

### Measurement of Short-Chain Fatty Acids

Colon contents were homogenized and centrifuged. The supernatant was mixed with an internal standard, ether and sulfuric acid (50%). Finally, the solution was centrifuged and the supernatant was used to quantify short-chain fatty acids (SCFA). SCFAs were detected using a gas chromatography-mass spectrometer (Agilent 8890) equipped with an Agilent 19091N-133I column (30 m × 250 μm × 0.25 μm). The detection parameters are: injection volume 1 μL; inlet temperature 250°C; ion source temperature 230°C; transfer line temperature 250°C, quadrupole temperature 150°C; split ratio 10:1; heating program: initial temperature 60°C, hold for 5 min, ramp to 110°C at 10°C/min, then ramp to 250°C at 35°C/min, hold for 1 min; carrier gas (helium) flow rate 1 mL/min.

### 16S rDNA Amplicon Sequencing Analysis of Colonic Microbiota

The procedures for total DNA extraction, amplification, sequencing of the V3-V4 region of the 16S rRNA gene, and construction of a sequencing library are illustrated in the Supplementary Methods. Downstream amplicon bioinformatic analyses were performed using EasyAmplicon v.1.023, as described by Qi et al. (31). Non-redundant sequences were de-noised into ASVs via the-unoise3 command of USEARCH (v.10.0). ASVs were classified using the Bayesian lowest common ancestor (BLCA) at the species level. Minimum count and low variance were used to filter the sequencing data. Principal coordinate analysis (PCoA) was performed to assess differences between groups using the analysis of similarity (ANOSIM) tool. Microbial gene pathways were predicted using the Phylogenetic Investigation of Communities by Reconstruction of Unobserved States 2 (PICRUSt2) pipeline v.2.3.0-b with default parameters. Differential abundance analysis of PICRUSt2-inferred MetaCyc pathways and microbial communities at the species level were performed using STAMP v.2.1.3 with a two-sided Welch’s *t*-test.

### Ultra-Performance Liquid Chromatography-Tandem Mass Spectrometry of Colon Content

Sample preparation, ultra-performance liquid chromatography-tandem mass spectrometry (UPLC-MS/MS) spectral acquisition conditions, and data analysis methods were as described in our previous study (10).

### Statistical Analysis

Data were statistically analyzed using IBM SPSS Statistics v.20.0 (Chicago, IL, United States). Results are expressed as means ± standard error of the mean (SEM). Differences between groups were compared using one-way analysis of variance (ANOVA), followed by Duncan’s test (equal variances assumed) or the Mann–Whitney U–test (equal variance not assumed). Differences was were considered statistically significant when *P*-value was ≤0.05.

## Results

### Effects of DSFS on Immobility Duration in Tail Suspension Test

**FIGURE 2:**
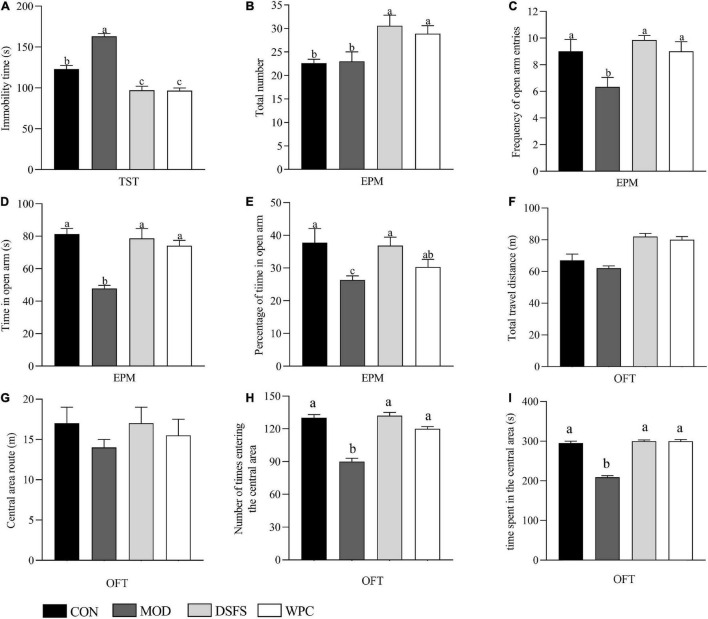
Effect of DSFS on tail suspension test (TST), elevated plus maze test (EPM), and open field test (OFT). **(A)** Immobility time of TST; **(B)** total number; **(C)** frequency of open arm entries; **(D)** time in open arm; **(E)** percentage of time in open arm; **(F)** total travel distance in open field; **(G)** travel distance in the central area of OFT; **(H)** frequency of reaching the central area of the open field; **(I)** time spent in the central area of the open field. Data are expressed as mean ± SEM (*n* = 10 per group). The values with different superscript letters in a column are significantly different (*P* < 0.05). The letter is the same, indicating no significant difference. CON, normal control group; MOD, model control group; DSFS, deoiled and dechlorogenic acid sunflower seeds group; WPC, whey protein group.

Compared to the CON group, a longer immobility duration was observed in the MOD group (*P* < 0.05, Figure 2A). DSFS and WPC administration markedly decreased the immobility duration relative to the MOD group (*P* < 0.05).

### Effects of DSFS on Anxiety-Like Behavior in an Elevated Plus-Maze Test

No statistical difference was observed in the total number of the elevated plus-maze in the CON and MOD groups, while the frequency of open arm entries, time in open arm, and percentage of time in open arm were lower in the MOD group than in the CON group (*P* < 0.05, Figure 2). These parameters were significantly improved after DSFS and WPC treatments.

### Effects of Depression/Anxiety-Like Behavior in Open Field Tests

The total distance of mice moving in the open field can reflect the autonomous activity of mice. The distance in the central area, the time and times of entering the central area not only reflect the autonomous exploration activities of mice, but also indirectly reflect the anxiety state of mice. As shown in Figures 2F–I, the total distance in the open field and the distance into the central field of the mice in the CON, DSFS, and WPC groups were not significantly different from those in the MOD group (*P* > 0.05). The number and time of entering the central area in the MOD groups were significantly lower than those in the CON group (*P* < 0.05). Compared with the MOD group, the DSFS and WPC group significantly increased the number and time of stress mice entering the central area (*P* < 0.05).

### Effects of DSFS on Oxidative Stress in Liver

**FIGURE 3:**
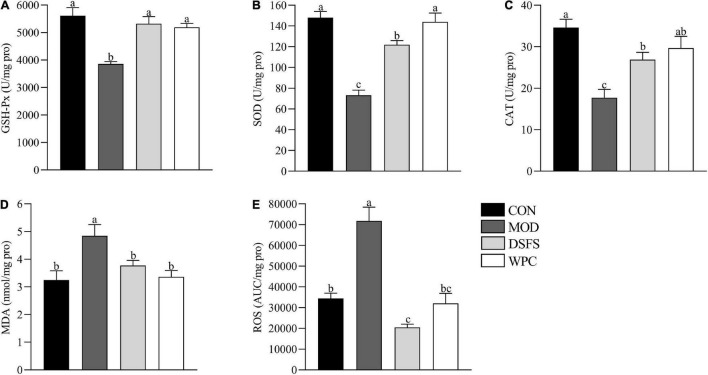
Effects of DSFS on a redox state in liver. **(A)** glutathione peroxidase (GSH-Px) activity levels; **(B)** superoxide dismutase (SOD) activity levels; **(C)** catalase (CAT) activity levels; **(D)** malondialdehyde (MDA) levels; **(E)** reactive oxygen species (ROS) levels. Data are expressed as mean ± SEM (*n* = 10 per group). The values with different superscript letters in a column are significantly different (*P* < 0.05). The letter is the same, indicating no significant difference. CON, normal control group; MOD, model control group; DSFS, deoiled and dechlorogenic acid sunflower seeds group; WPC, whey protein group.

Figure 3 shows the influence of DSFS on the liver redox state. In contrast to the CON group, GSH-Px, SOD, and CAT activities were lower in the MOD group (*P* < 0.05). However, elevated levels of MDA and ROS were found in the MOD group compared with those in the CON group (*P* < 0.05). In contrast, both DSFS and WPC diets elevated GSH-Px, SOD, and CAT activities, and restored MDA and ROS to normal levels relative to the MOD group (*P* < 0.05).

### Effects of DSFS on Plasma Corticosterone and Hippocampal 5-Hydroxytryptamine Levels

**FIGURE 4:**
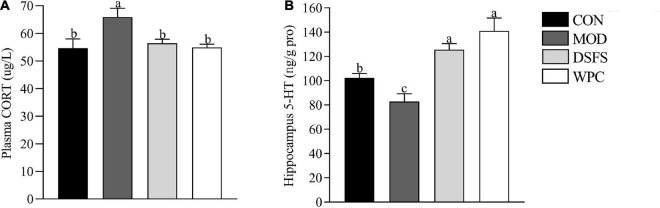
Effects of DSFS on corticosterone (CORT) **(A)** levels in plasma and 5-hydroxytryptamine (5-HT) **(B)** levels in the hippocampus. Data are expressed as mean ± SEM (*n* = 10 per group). The values with different superscript letters in a column are significantly different (*P* < 0.05). The letter is the same, indicating no significant difference. CON, normal control group; MOD, model control group; DSFS, deoiled and dechlorogenic acid sunflower seeds group; WPC, whey protein group.

Plasma corticosterone levels were increased in the MOD group (*P* < 0.05) and found to be decreased after DSFS and WPC administration (*P* < 0.05, Figure 4A).

Long-term stress led to a decrease in 5-HT levels in the MOD group relative to those in the CON group (*P* < 0.05, Figure 4B). 5-HT level in the DSFS and WPC groups were higher than those in the MOD group (*P* < 0.05).

### Effects of DSFS on Intestinal Mucosal Barrier Function Histology

**FIGURE 5:**
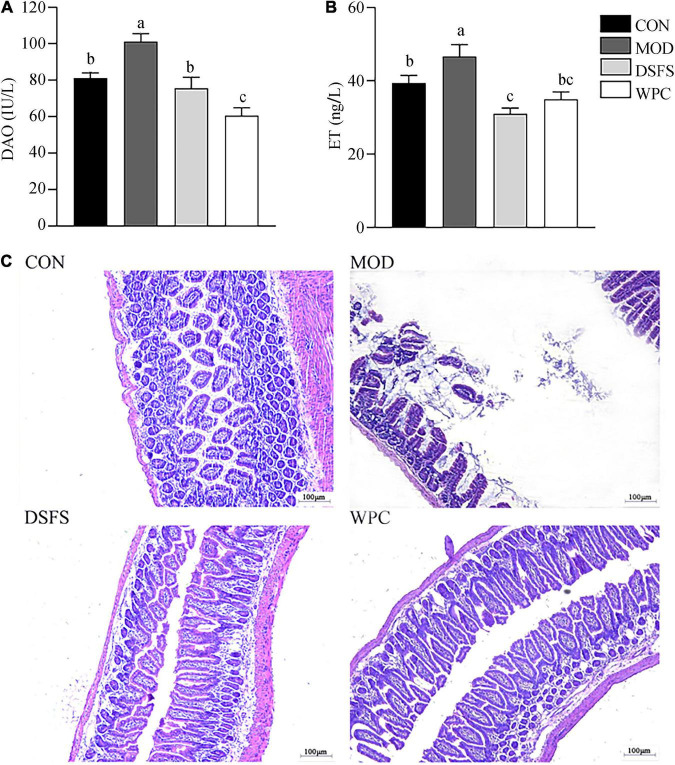
Effects of DSFS on plasma diamine oxidase (DAO) **(A)**, endotoxin (ET) **(B)**, and the histology of the ileum **(C)** in stressed mice. Data are expressed as mean ± SEM (*n* = 10 per group). The values with different superscript letters in a column are significantly different (*P* < 0.05). The letter is the same, indicating no significant difference. CON, normal control group; MOD, model control group; DSFS, deoiled and dechlorogenic acid sunflower seeds group; WPC, whey protein group.

As shown in Figures 5A,B, chronic stress induced a significant increase in DAO and ET levels in MOD mice compared with CON mice (*P* < 0.05). In addition, the DSFS and WPC diets downregulated DAO and ET levels compared to those in MOD mice (*P* < 0.05).

The intestinal villi were dense and well-arranged in CON mice (Figure 5C). In contrast, their tissue morphology was visibly disrupted, and the villi were fractured in MOD mice. The cells showed local dissolution, and the nuclei were wrinkled, deformed, and deeply stained in stressed mice. Furthermore, villi were dense and well-arranged after DSFS and WPC administration.

### Effects of DSFS on mRNA Expression in Colon

**FIGURE 6:**
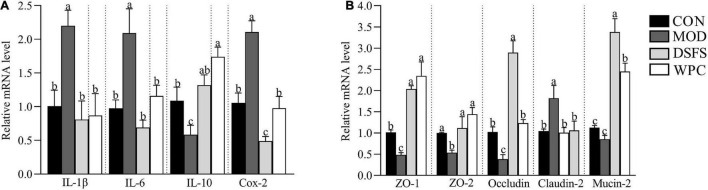
Effects of DSFS on the expression of inflammatory cytokines **(A)** and colonic tight junction proteins **(B)**. Data are expressed as mean ± SEM (*n* = 10 per group). The values with different superscript letters in a column are significantly different (*P* < 0.05). The letter is the same, indicating no significant difference. CON, normal control group; MOD, model control group; DSFS, deoiled and dechlorogenic acid sunflower seeds group; WPC, whey protein group.

Compared with the CON group, the relative mRNA expression of *IL-1*β, *IL-6*, and cyclooxygenase-2 genes was significantly increased in the MOD group (*P* < 0.05, Figure 6A), whereas a significant downregulation in *IL-10* mRNA expression was found in the MOD group (*P* < 0.05). Moreover, DSFS and WPC diets reversed the effects induced by chronic stress.

In addition, we measured the expression of mucosal barrier-related genes (Figure 6B). Compared to the CON group, downregulation of zonula occludens (ZO) 1, *ZO-2*, and occludin mRNA expression was observed in stressed mice (*P* < 0.05), whereas claudin-2 mRNA expression was upregulated in stressed mice (*P* < 0.05), indicating increased intestinal permeability in these mice. Compared with stressed mice, these changes were restored in the DSFS and WPC groups.

### Effects of DSFS on Short-Chain Fatty Acids in Colon Content

**FIGURE 7:**
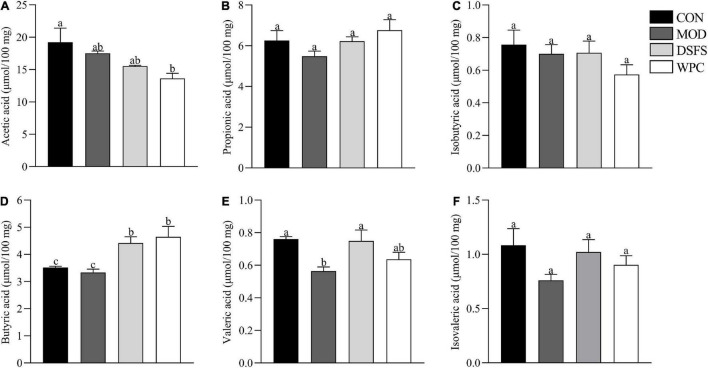
Effects of DSFS on SCFA in the colon content. **(A)** acetic acid levels; **(B)** propionic acid levels; **(C)** isobutyric acid levels; **(D)** butyric acid levels; **(E)** valeric acid levels; **(F)** isovaleric acid levels. Data are expressed as mean ± SEM (*n* = 10 per group). CON, normal control group; MOD, model control group; DSFS, deoiled and dechlorogenic acid sunflower seeds group; WPC, whey protein group. The values with different superscript letters in a column are significantly different (*P* < 0.05). The letter is the same, indicating no significant difference.

Compared with CON mice, valeric acid levels were lower in MOD mice (*P* < 0.05, Figure 7). In contrast, butyric and valeric acid levels were markedly elevated after DSFS treatment (*P* < 0.05). Additionally, increased levels of butyric acid were observed in WPC mice compared to those in MOD mice (*P* < 0.05).

### DSFS Modulates Gut Microbiota Structure in Mice

**FIGURE 8:**
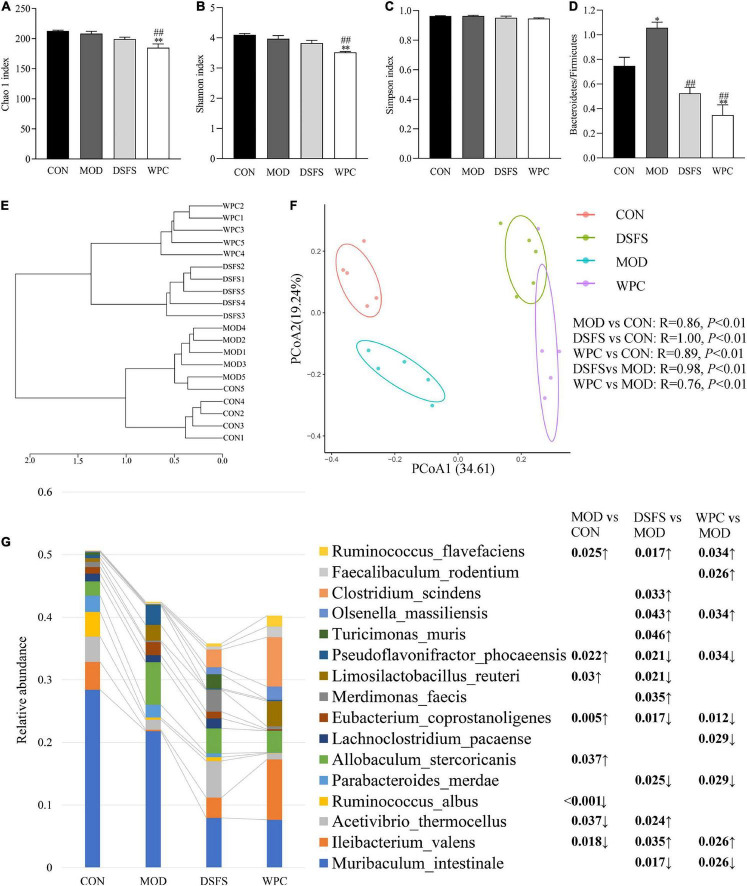
Effects of DSFS on gut microbiota structure in stressed mice. **(A–C)** α diversity. **(D)** The ratio of Bacteroidetes to Firmicutes. **(E)** Dendrogram of Hierarchical clustering analysis with distance measures based upon the Bray–Curtis index. **(F)** β diversity, the principal coordinate analysis (PCoA) was performed based on OTUs using the analysis of similarity (ANOSIM) tool, based upon a Bray–Curtis similarity matrix. **(G)** Profile of dominant species (The relative abundance in at least one sample is greater than 1%) and the FDR adjusted *P*-value of inter group difference analysis based on two sided Welch’s *t*-test. Data are expressed as mean ± SEM (*n* = 5 per group). *P < 0.05, ^**^P < 0.01, compared with the CON group; ^##^*P* < 0.01, compared with the MOD group. CON, normal control group; MOD, model control group; DSFS, deoiled and dechlorogenic acid sunflower seeds group; WPC, whey protein group.

To further assess the effects of DSFS on gut microbiota structures in mice, we measured the gut microbiota in terms of colon content by *16S* rDNA PCR amplicon sequencing. Figures 8A–C illustrates the α diversity of the colonic microbiota. Differences in the Chao 1, Shannon, and Simpson indexes were not significant in the CON, MOD, and DSFS groups (*P* > 0.05, Figures 8A–C). Additionally, the Chao 1 and Shannon indexes in the WPC group were notably lower than those in the other three groups, suggesting that species richness and diversity were decreased by WPC treatment. Furthermore, chronic stress induced a significant increase in the ratio of Bacteroidetes to Firmicutes, which was remarkably attenuated by DSFS and WPC supplementation (Figure 8D). The dendrogram of the hierarchical clustering analysis suggested that the microbiome in each group was relatively similar, and the differences between the MOD and DSFS groups were significant (Figure 8E). The PCoA of β diversity, based on a Bray–Curtis similarity matrix, demonstrated that different gut microbiota structures existed among the four groups (ANOSIM, *P* < 0.05, Figure 8F). This change suggested that chronic stress disturbs gut microflora. Moreover, the gut microbiota of DSFS- and WPC-treated mice differed from that of the stressed mice.

As shown in Figure 8G, CUMS significantly increased the relative contents of intestinal *Pseudoflavonifractor phocaeensis* (*P* < 0.05) and *Eubacteium coprostanoligenes* (*P* < 0.01), while the DSFS and WPC-treated groups significantly decreased relative to the MOD group (FDR < 0.05). *Limosilactobacillus reuteri* was significantly up-regulated (*P* < 0.05) in the MOD group, and relatively down-regulated (*P* < 0.05) in the DSFS-treated group. CUMS caused a significant down-regulation of intestinal *I. valens* (*P* < 0.05), which was significantly up-regulated in DSFS and WPC-treated mice relative to MOD mice (*P* < 0.05). Meanwhile, both treatments significantly up-regulated *Olsenella massiliensis* and down-regulated the relative content of *Parabacteroides merdae* (*P* < 0.05). DSFS treatment also specifically up-regulated the relative content of *Clostridium massiliensis*, *Turicimonas muris* and *Merdimonas faecis* (*P* < 0.05).

**FIGURE 9:**
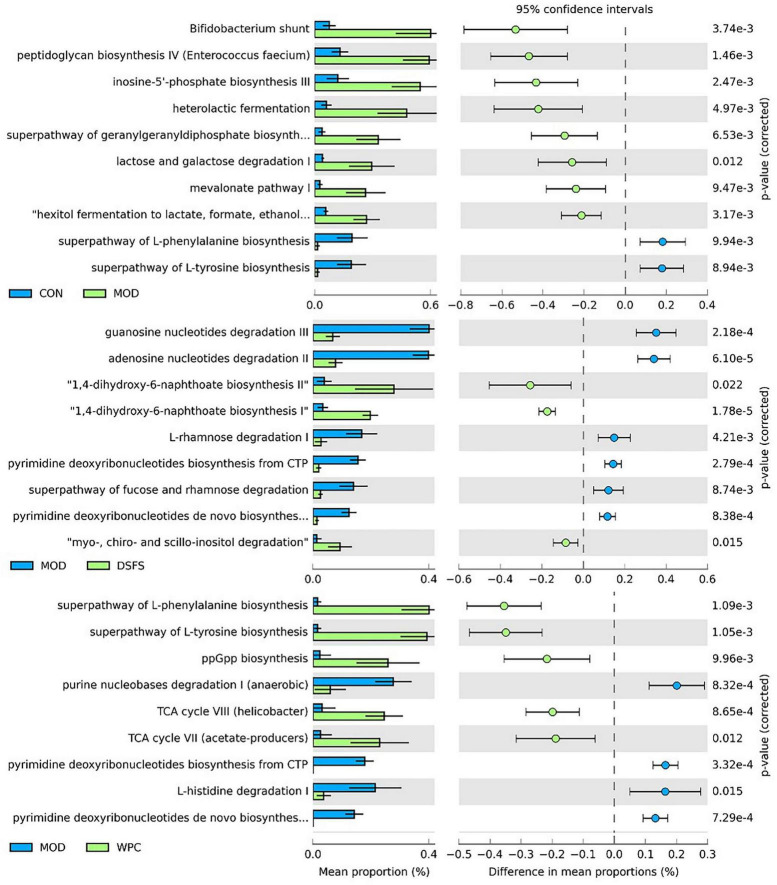
Significant differences (FDR-adjusted *P*-value < 0.05) between groups in MetaCyc gene pathways predicted by PICRUSt2. CON, normal control group; MOD, model control group; DSFS, deoiled and dechlorogenic acid sunflower seeds group; WPC, whey protein group.

Furthermore, we observed that the MetaCyc gene pathways were significantly different between the groups (FDR-adjusted *P*-value < 0.05, Figure 9). Ten pathways were identified for comparison between the CON and MOD groups, including Bifidobacterium shunt, peptidoglycan biosynthesis IV (*Enterococcus faecium*), inosine-5’-phosphate biosynthesis III, and the superpathway of L-tyrosine biosynthesis. The mean proportion of the eight pathways was significantly increased, while that of the two pathways was significantly decreased in the MOD group relative to the CON group. Nine pathways were significantly altered in the DSFS group compared to the MOD group, with three pathways being significantly increased (1,4-dihydroxy-6-naphthoate biosynthesis II, 1,4-dihydroxy-6-naphthoate biosynthesis II, myo-, chiro-, and scillo-inositol degradation) and six pathways being decreased (guanosine nucleotide degradation III, adenosine nucleotides degradation II, L-rhamnose degradation I, pyrimidine deoxyribonucleotides biosynthesis from CTP, superpathway of fucose, and rhamnose degradation).

### DSFS Modulates Metabolites of Colon Contents

**FIGURE 10:**
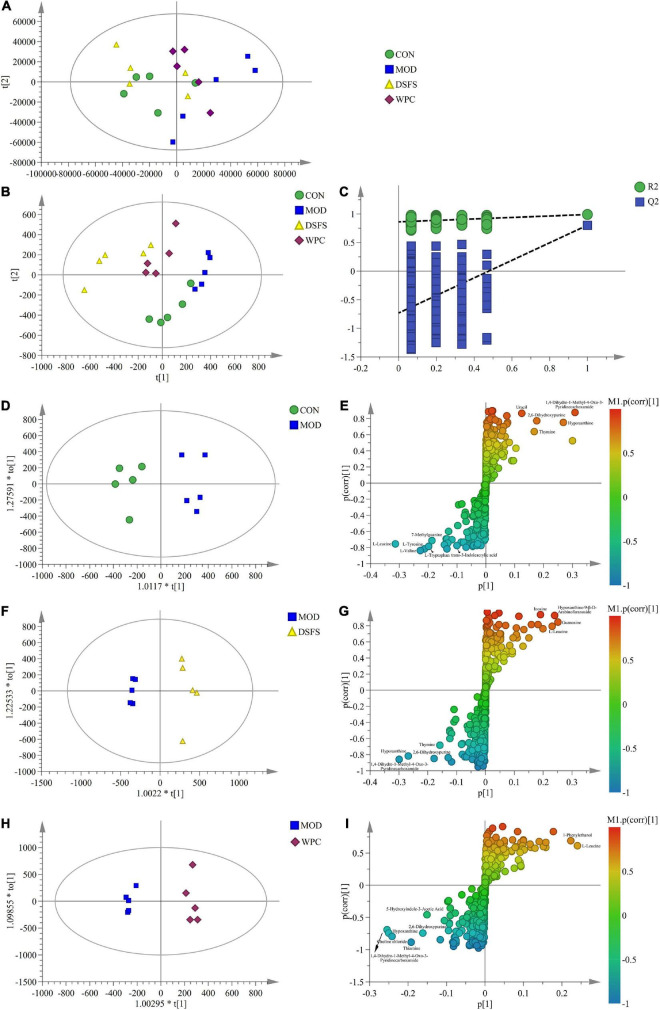
Extensive targeted metabolome and multivariate statistical analysis in colon content. **(A)** PCA plot (R^2^X = 0.925, Q^2^ = 0.507). **(B)** PLS-DA plot (R^2^X = 0.908, R^2^Y = 0.987, Q^2^ = 0.774). **(C)** PLS-DA validation plot. **(D,F,H)** OPLS-DA plot [**(D)**: R^2^X = 0.691, R^2^Y = 0.932, Q^2^ = 0.572, **(F)**: R^2^X = 0.558, R^2^Y = 0.974, Q^2^ = 0.878, **(H)**: R^2^X = 0.532, R^2^Y = 0.985, Q^2^ = 0.713]. **(E,G,I)** S-plot (*n* = 5 per group). CON, normal control group; MOD, model control group; DSFS, deoiled and dechlorogenic acid sunflower seeds group; WPC, whey protein group.

Principal component analysis (PCA), partial least squares discriminant analysis (PLS-DA), and orthogonal PLS-DA (OPLS-DA) models were established to explore the metabolic characteristics of the mice (Figure 10). The PCA plot showed that the samples in the MOD group were significantly separated from the remaining three cohorts, suggesting that there were differences between MOD-treated animals and the remaining three groups. In addition, there was a partial overlap between the sample points of the DSFS, WPC, and CON groups, indicating that DSFS and WPC were able to restore metabolic disorders to a normal state. The PLS-DA plot further verified that the MOD group was significantly different from the other three groups, and the permutation verification plot demonstrated that the model verification was reasonable.

Furthermore, the OPLS-DA models revealed significant differences between the MOD group and the other three groups. The corresponding S-plot was used to screen for potential biomarkers. Table 1 presents detailed information regarding the differential metabolites (VIP > 1.00 and *P* < 0.05).

**TABLE 1:** Effect of DSFS on metabolites of colon content in mice.

Compounds	MOD vs. CON			DSFS vs. MOD			WPC vs. MOD		
	VIP	FC	Change	VIP	FC	Change	VIP	FC	Change
1,4-Dihydro-1-Methyl-4-Oxo-3-Pyridinecarboxamide	7.20	1.51	↑^#^	7.00	0.49	↓*	5.99	0.72	↓*
2,6-Dihydroxypurine	4.09	1.41	↑^#^	4.18	0.57	↓*	3.80	0.74	↓*
Hypoxanthine				6.30	0.44	↓*	5.85	0.67	↓*
Uracil	2.88	1.65	↑^#^	2.78	0.45	↓**	2.22	0.76	↓*
7-Methylguanine	4.37	0.77	↓^#^	4.28	1.42	↑**			
Thymine				3.71	0.43	↓*			
6-Methylmercaptopurine	2.73	0.68	↓^#^	2.52	1.65	↑*			
L-Leucine	7.29	0.83	↓^#^	5.41	1.15	↑*			
L-Valine	5.29	0.73	↓^##^						
L-Tyrosine	4.93	0.79	↓^#^						
L-Tryptophan	4.64	0.56	↓^#^						
Indoleacetaldehyde				1.13	3.65	↑**	1.09	2.55	↑**
(E)-3-Indoleacrylic acid	2.36	0.53	↓^#^						
Methoxyindoleacetic acid	1.04	0.58	↓^#^						
Azelaic acid	1.77	1.26	↑^#^	1.88	0.65	↓*	2.72	0.60	↓*
*N*-acetylhistamine	1.70	1.61	↑^#^	1.57	0.48	↓*	1.55	0.66	↓*
Hydrocinnamic acid	1.39	2.47	↑^#^	1.21	0.35	↓*	1.89	0.16	↓*
1-Phenylethanol				4.66	1.86	↑*	5.25	1.79	↑*
2-(Formylamino)Benzoic acid	3.11	0.71	↓^#^	3.11	1.61	↑*			
D-Mannose							2.33	3.20	↑*
D-Fructose							2.17	2.92	↑*
Thiamine				3.06	0.16	↓*	4.49	0.04	↓*

*^#^Represents a significant difference compared with CON group (P < 0.05). ^##^Represents a highly significant difference compared with the CON group (P < 0.01).*

*Represents a significant difference compared with the MOD group (P < 0.05). **Represents a highly significant difference compared with the MOD group (P < 0.01).

*↑Indicates a significant increase. ↓Indicates a significant decrease.*

Fifteen metabolites were significantly altered in the MOD group compared to those in the CON group, with nine metabolites being downregulated and six being upregulated (Table 1). These metabolites were mainly represented by significantly lower concentrations of L-trp, L-leucine, L-valine, L-tyrosine, trans-3-indoleacrylic acid, methoxyindoleacetic acid, 7-methylguanine and 6-methylmercaptopurine, and significantly higher concentrations of 1, 4-dihydro-1-methyl-4-oxo-3-pyridinecarboxamide, 2, 6-dihydroxypurine, uracil, and azelaic acid. Compared with the MOD group, 15 and 12 metabolites were significantly altered in the DSFS and WPC groups, respectively. The DSFS intervention significantly reduced 2, 6-dihydroxypurine, hypoxanthine, uracil, thymine, azelaic acid, hydrocinnamic acid, and thiamine levels in stressed mice and increased 7-methylguanine, 6-methylmercaptopurine, L-leucine, and indoleacetaldehyde levels. These endogenous metabolites are mainly involved in 16 metabolic pathways, including Trp metabolism; aminoacyl-tRNA biosynthesis; valine, leucine and isoleucine biosynthesis; phenylalanine, tyrosine, and Trp biosynthesis; and thiamine metabolism.

### Correlation Analysis of Gut Microbiota and Biochemical Indicators

**FIGURE 11:**
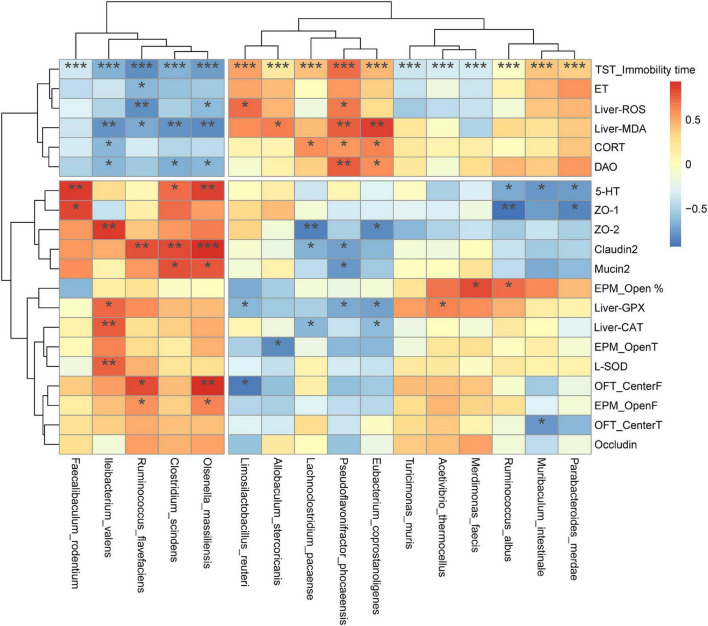
Spearman correlation analysis between colonic bacterial species and biochemical indicators. The x-axis and y-axis of the heat map are gut microbiota, biochemical and behavioral indicators respectively, and the *R*-value and *P*-value are obtained by calculation. The color of the bar shows the correlation coefficient. *P < 0.05, **P < 0.01, ***P < 0.001.

Figure 11 presents a correlation heat map of gut microbiota and biochemical indicators. Four species of gut bacteria that were up-regulated by DFSF, *I. valens, Ruminococcus flavefaciens, Clostridium scindens*, and *O. massiliensis* were significantly negatively associated with adverse depression markers, including TST immobility time, plasma endotoxin, and liver ROS and MDA, hypothalamic CORT and plasma DAO. They were positively correlated with the rest of the indicators that were positively associated with depression. Among the remaining strains, three down-regulated by DSFS and/or WPC intervention, *Lachnoclostridium pacaense*, *P. phocaeensis*, and *Eubacteriumcor prostanoligenes*, were positively correlated with other depressive factors other than plasma endotoxin. They are typical Depression-related species. In addition, it is worth noting that the immobility time of TST was significantly associated with all species of bacteria analyzed (*P* < 0.001).

**FIGURE 12:**
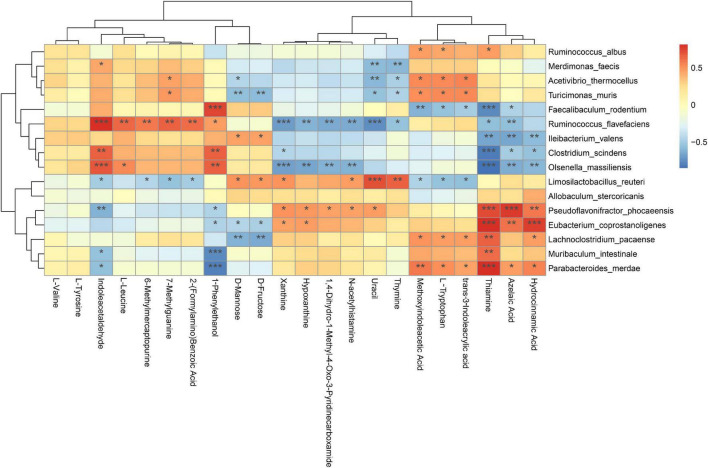
Spearman correlation analysis of species and metabolites of mouse colonic microbiota. Red: positive correlation, blue: negative correlation; *P < 0.05, **P < 0.01, ***P < 0.001.

To explore the potential relationships between the gut microbiome changes and metabolic products, a correlation matrix was generated using Spearman correlation. Figure 12 shows that the analyzed species were clustered into three clusters, where the five DSFS antidepressant-responsive species revealed by Figure 11 are in the heatmap cluster II. Colonic metabolites were clustered into two clusters, and those of the first cluster were positively correlated with cluster II species of the gut microbiota. Among them, Indoleacetaldehyde was specially positively correlated with *R. flavefaciens*, *C. scindens*, and *Olsenella massiiensis*, and 1-Phenylethanol was positively correlated with four bacteria except *I. valens*. Three depression-related species, *P. phocaeensis*, *Eubacterium coprostanoligenes* and *L. pacaense*, *Muribaculum intestinale*, and *P. merdae* were negatively correlated with Thiamine, Azelaic aci, and Hydrocinnamic acid, especially Thiamine.

## Discussion

In the present study, mice subjected to chronic stress exhibited longer durations of immobility, and less entry times and time spent in the middle of the open field indicating the prevalence of depression-like behaviors. In agreement with these results, chronic stress resulted in a longer immobility duration in tail suspension test (32). In contrast, DSFS and WPC diets decreased the immobility time and increased the number of explorations and time spent in the central area of the open field, suggesting antidepressant-like activity. Similar results were observed in a previous study in which immobility time was normalized after α-lactalbumin treatment (6). Furthermore, chronic stress induced anxiety-like behavior in mice, whereas DSFS and WPC diets effectively restored depression-like behaviors to normal levels. This outcome is consistent with that of Orosco et al. (33), who found that α-lactalbumin prevented a decrease in the percentage of time spent in open arms in elevated plus maze test. Overall, the behavioral results indicated that DSFS exerted a beneficial impact in improving depression- and anxiety-like behaviors.

It was found that patients with depression are accompanied by visible symptoms of intestinal dysfunction, and the severity is positively correlated with the degree of depression (34). For mice, CUMS destroys the intestinal mucosa and enhances intestinal permeability (35). The destruction of the integrity of the mucosal barrier might be related to the decrease in tight junction protein expression and inhibition of mucosal layer function via the reduction in goblet cell numbers (14). Moreover, tissue morphology and villi were destroyed. It has been reported that long-term stress disrupts gut barrier function and causes serious intestinal inflammatory responses (36). Profound epithelial structural disruption and significant downregulation of tight junction protein expression were observed in stressed mice. In contrast, the expression of mucin-2 and tight junction proteins was effectively upregulated after DSFS and WPC administration, which might have contributed to decrease expression of *IL-1*β and *IL-6* expression. This mechanism may be related to an increase in the mucus layer (37). GSH-Px, SOD, and CAT activities were decreased, whereas, MDA and ROS levels were elevated in liver of stressed mice. These observations were in agreement with a study in which chronic mild stress caused elevated production of ROS and MDA and a reduction in GSH-Px and CAT levels in the liver (38). The DSFS and WPC diets restored oxidative stress markers and key antioxidant enzymes to normal levels. DSFS might reduce ROS generation by scavenging radicals, thereby attenuating the damage to serotoninergic neurons induced by MDA (39). A similar finding revealed that α-lactalbumin treatment decreased MDA levels and normalized GSH levels (40). More importantly, CUMS reduced 5-HT levels in the hippocampus, which were restored by chronic administration of DSFS and WPC. In our previous study, WPC and DSFS significantly increased plasma Trp and Trp/LNAAs levels (10). This might be because dietary DSFS and WPC, as direct sources of Trp, increase the ratio of Trp to large neutral amino acids, thereby enhancing 5-HT levels in the brain.

Accumulating evidence suggests that alterations in the gut microbiota and microbe-derived metabolites play a key role in the pathophysiology of depression via the brain–gut–microbiota axis (41). It was found that CUMS treatment induced changes in the β diversity of gut microbiota and up-regulation of plasma intestinal mucosal barrier markers, ET and DAO, resulting in depression-like behavioral changes, which also supports the theory that the brain-gut axis is associated with depression. It was suggested that alternations of gut microbiome and metabolites was potential mediators in efficiency of antidepressants treatment (42). In this study, DSFS and WCP intervention showed antidepressant effects, which were related to the regulation of intestinal flora and the enhancement of mucosal barrier. The key species promoted by the DSFS were *I. valens*, *R. flavefaciens*, *C. scindens* and *Olsenella massiliensis*. *I. valens* showed potential for modulation of intestinal inflammation and immunity by modulating intestinal immune gene expression of RORγT, IL-17A, IL-17F, ReglIlγ, Relmß, and/or Defβ (43). *R. flavefaciens* are well-known cellulolytic bacteria involved in the butyrate metabolic pathway (44), and its upregulation may be related to the promotion of butyrate production in the gut microbiota by DSFS. *C. scindens* is actively involved in the conversion of cholate to deoxycholate, which may be important in treatment of disease associated with increased colonic secondary bile acids (45). *O. massiliensis* is newly found strictly anaerobic bacterium which is responsible for the production of lactic, formic, and acetic acids as fermentation products from glucose in gut (46, 47). Taken together, these bacteria may enhance the mucosal barrier either directly or by promoting SCFA production. DSFS treatment mainly down-regulated *P. phocaeensis* and *Eubacter-iumcor prostanoligenes.* There have been no reports of them having negative health effects. In addition, relative abundance of *Lactobacillus reuteri* (*Limosilactobacillus reuteri*) was significantly increased in the MOD group relative to the CON group, but decreased after DSFS treatment. It is a potential species that causes depression in mice (48), which is consistent with the results of this study.

Alterations in gut metabolites may be the main reason why microbiota remodeling affects behavior. Indoleacetaldehyde is a key gut microbiota metabolite positively associated with *C. scindens* and *O. massiliensis*. It is a gut bacterial metabolite of tryptophan, an aryl hydrocarbon receptor (AhR) involved in gut barrier protection by activating IL-22 (49). Several types of brain cells, including neurons, astrocytes, and microglial cells, express AhR (50). Multiple central nervous system illnesses have been linked to a decrease in circulating AhR agonist levels originating from the gut microbiota (51). Both DSFS and WPC are typical tryptophan-rich protein sources, and some undigested residues enter the posterior digestive tract and may be metabolized by intestinal microbiota to produce indole derivatives, including Indoleacetaldehyde. The intestinal L-tryptophan of mice in the MOD group was depleted, and its metabolites (E)-3-indoleacrylic acid and methoxyindoleacetic acid were also significantly decreased, indicating that the intestinal tract could not meet the demand for AhR ligands during stress. When DSFS and WPC are used as dietary proteins, they provide tryptophan that enhances gut-derived AhR ligands such as indoleacetaldehyde. 1-Phenylethanol, a sweet, acetophenone, and fresh-tasting compound, was another DSFS-induced compound that was significantly positively correlated with *C. scindens* and *O. massiliensis*. Its isomers, 2-phenylethanol, was reported to elicit neuropsychological effects that alter the behavior of mice and may also elicit anti-depressive effects (52).

Altered purine circulating metabolic activity is associated with depression-related systemic responses, such as abnormal inflammation and aggravation of oxidative stress (53). Hypoxanthine and 2,6-dihydroxypurine are important products of purine metabolism. In this study, xanthine level were significantly higher in stressed mice, which might be due to increased activity of xanthine oxidase induced by stress and by the increased proportion of hypoxanthine metabolism to xanthine (54). Xanthine is positively correlated with the degree of oxidative stress, and elevated levels increase ROS production, leading to increased oxidative stress. A previous study reported that xanthine oxidase and xanthine levels are increased in patients with major depression (54), which is consistent with our observations. Under stress, the expression and activity of xanthine oxidase are enhanced, which in turn leads to excessive production of xanthine and uric acid. DSFS and WPC supplementation reduced hypoxanthine and xanthine levels in stressed mice, suggesting that they may modulate the purine metabolic pathway by regulating xanthine oxidase activity.

Uracil is a product of pyrimidine metabolism and the pyrimidine metabolic pathway is involved in the body’s energy metabolism through various reactions. In the present study, uracil levels were increased in stressed mice, indicating stress-induced abnormalities in pyrimidine metabolism. Uracil levels in the DSFS- and WPC-administered groups were significantly lower than those in the MOD group. Xu et al. (55) also observed disturbances involving uracil metabolism in subjects with bipolar disorder. In addition, pyrimidine metabolism has been reported to affect the normal development and function of the central nervous system. Abnormal pyrimidine metabolism causes apoptosis and mitochondrial damage in brain cells, leading to central nervous system dysfunction (56). These above results suggest that DSFS might improve stress in mice by regulating pyrimidine metabolic pathways.

## Conclusion

The DSFS diet alleviated multiple depression-like symptoms in CUMS-induced mice. Intaking of feed with DSFS as protein source resulted in the modulation of gut microbiota and Trp metabolism, alleviation of oxidative stress and inflammatory responses, and improvement in intestinal mucosal barrier function. There was a strong correlation between gut microbiota and colonic tryptophan metabolism. These findings provide new insights for the development of sunflower seed protein-based foods. Seed protein-based foods may represent a promising new therapeutic tool to improve depression symptoms.

## Data Availability Statement

The datasets presented in this study can be found in online repositories. The names of the repository/repositories and accession number(s) can be found in the article/Supplementary Material.

## Ethics Statement

The animal study was reviewed and approved by Jiangnan University Animal Care and Use Ethics Committee.

## Author Contributions

XL: conducted the work, analyzed the data, and wrote the manuscript. CQ: acquired the data and revised the work. LJ, MS, and JZ: provided technical support and experimental materials. JS: designed and revised the work. All authors contributed to the article and approved the submitted version.

## Conflict of Interest

XL, JZ, MS, and LJ were employed by Qiaqia Food Co., Ltd. The remaining authors declare that the research was conducted in the absence of any commercial or financial relationships that could be construed as a potential conflict of interest.

## Publisher’s Note

All claims expressed in this article are solely those of the authors and do not necessarily represent those of their affiliated organizations, or those of the publisher, the editors and the reviewers. Any product that may be evaluated in this article, or claim that may be made by its manufacturer, is not guaranteed or endorsed by the publisher.

## References

[1] Lackamp J Schlachet R Sajatovic M. Assessment and management of major depressive disorder in older adults. *Psychiatria Danubina.* (2016) 28:95–8. 27663815

[2] Abehiguchi N Uchida S Yamagata H Higuchi F Hobara T Hara K Hippocampal sirtuin 1 signaling mediates depression-like behavior. *Biol Psychiatry.* (2016) 80:815–26. 10.1016/j.biopsych.2016.01.009 27016384

[3] Yan K Chen YB Wu JR Li KD Cui YL. Current rapid-onset antidepressants and related animal models. *Curr Pharm Des.* (2018) 24:2564–72. 10.2174/1381612824666180727115222 30051782

[4] Wong PH Ong YP. Acute antidepressant-like and antianxiety-like effects of tryptophan in mice. *Pharmacology.* (2001) 62:151–6. 10.1159/000056088 11287816

[5] Hudson C Hudson S MacKenzie J. Protein-source tryptophan as an efficacious treatment for social anxiety disorder: a pilot study. *Can J Physiol Pharmacol.* (2007) 85:928–32. 10.1139/Y07-082 18066139

[6] Yu VO Peuhkuri K Bäckström P Sihvola N Pilvi T Korpela R. The effects of native whey and α-lactalbumin on the social and individual behaviour of C57bl/6j Mice. *Br J Nutr.* (2013) 110:1336–46. 10.1017/S0007114513000238 23507076

[7] Scrutton H Carbonnier A Cowen PJ Harmer CJ. Effects of alpha-lactalbumin on emotional processing in healthy women. *J Psychopharmacol.* (2007) 21:519–24. 10.1177/0269881106075271 17446205

[8] Ahmed R Eldenshary E Nada S Asaad G Arafa N Farid O. Pharmacological study of the possible antidepressant activity of whey protein isolate in mice. *Aust J Basic Appl Sci.* (2011) 5:2649–59.

[9] VolicerL. Can dietary intervention help in management of problem behaviors in dementia? *J Nutr Health Aging.* (2009) 13:499–501. 10.1007/s12603-009-0100-3 19536418

[10] Lu X Ce Q Jin L Zheng J Sun M Tang X Deoiled sunflower seeds ameliorate depression by promoting the production of monoamine neurotransmitters and inhibiting oxidative stress. *Food Funct.* (2021) 12:573–86. 10.1039/D0FO01978J 33367360

[11] Rothhammer V Mascanfroni ID Bunse L Takenaka MC Kenison JE Mayo L Type I interferons and microbial metabolites of tryptophan modulate astrocyte activity and central nervous system inflammation via the Aryl hydrocarbon receptor. *Nat Med.* (2016) 22:586–97. 10.1038/nm.4106 27158906 PMC4899206

[12] Dokalis N Prinz M. Resolution of neuroinflammation: mechanisms and potential therapeutic option. *Semin Immunopathol.* (2019) 41:699–709. 10.1007/s00281-019-00764-1 31705317

[13] Muku GE Murray IA Espín JC Perdew GH. Urolithin A is a dietary microbiota-derived human Aryl hydrocarbon receptor antagonist. *Metabolites.* (2018) 8:86–103. 10.3390/metabo8040086 30501068 PMC6315438

[14] Wei L Li Y Tang W Sun Q Chen L Wang X Chronic unpredictable mild stress in rats induces colonic inflammation. *Front Physiol.* (2019) 10:1228. 10.3389/fphys.2019.01228 31616319 PMC6764080

[15] Jang H-M Lee H-J Jang S-E Joo HM Dong-Hyun K. Evidence for interplay among antibacterial-induced gut microbiota disturbance, neuro-inflammation, and anxiety in mice. *Mucosal Immunol.* (2018) 11:1386–97. 10.1038/s41385-018-0042-3 29867078

[16] Wong ML Inserra A Lewis MD Mastronardi CA Leong L Choo J Inflammasome signaling affects anxiety– and depressive-like behavior and gut microbiome composition. *Mol Psychiatry.* (2016) 21:797–805. 10.1038/mp.2016.46 27090302 PMC4879188

[17] Wang Q Jia M Zhao Y Hui Y Pan J Yu H Supplementation of sesamin alleviates stress-induced behavioral and psychological disorders via reshaping the gut microbiota structure. *J Agric Food Chem.* (2019) 67:12441–51. 10.1021/acs.jafc.9b03652 31674783

[18] Anderson G Seo M Berk M Carvalho AF Maes M. Gut permeability and microbiota in Parkinson’s disease: role of depression, tryptophan catabolites, oxidative and nitrosative stress and melatoninergic pathways. *Curr Pharm Des.* (2016) 22:6142–51. 10.2174/1381612822666160906161513 27604608

[19] Bhatt S Nagappa AN Patil CR. Role of oxidative stress in depression. *Drug Discov Today.* (2020) 25:1270–6. 10.1016/j.drudis.2020.05.001 32404275

[20] Lucca G Comim CM Valvassori SS Gz R Vuolo F Petronilho F Effects of chronic mild stress on the oxidative parameters in the rat brain. *Neurochem Int.* (2009) 54:358–62. 19171172 10.1016/j.neuint.2009.01.001

[21] Lindqvist D Dhabhar FS James SJ Hough CM Jain FA Bersani FS Oxidative stress, inflammation and treatment response in major depression. *Psychoneuroendocrinology.* (2017) 76:197–205. 10.1016/j.psyneuen.2016.11.031 27960139 PMC5272818

[22] Bakunina N Pariante CM Zunszain PA. Immune mechanisms linked to depression via oxidative stress and neuroprogression. *Immunology.* (2015) 144:365–73. 10.1111/imm.12443 25580634 PMC4557673

[23] Pickardt C Neidhart S Griesbach C Dube M Knauf U Kammerer DR Optimisation of mild-acidic protein extraction from defatted sunflower (*Helianthus Annuus* L.) Meal. *Food Hydrocolloids.* (2009) 23:1966–73. 10.1016/j.foodhyd.2009.02.001

[24] Ren H Liu TC Lu Y Zhang K Xu Y Zhou P A comparison study of the influence of milk protein versus whey protein in high-protein diets on adiposity in rats. *Food Funct.* (2021) 12:1008–19. 10.1039/d0fo01960g 33502407

[25] Reeves EP Nielsen F Fahey G. Ain-93 purified diets for laboratory rodents: final report of the American institute of nutrition ad hoc writing committee on the reformulation of the Ain-76a rodent diet. *J Nutr.* (1993) 123:1939–51. 10.1093/jn/123.11.1939 8229312

[26] O’Leary OF Cryan JF. Towards translational rodent models of depression. *Cell Tissue Res.* (2013) 354:141–53. 10.1007/s00441-013-1587-9 23525777

[27] Mineur YS Belzung C Crusio WE. Effects of unpredictable chronic mild stress on anxiety and depression-like behavior in mice. *Behav Brain Res.* (2006) 175:43–50. 17023061 10.1016/j.bbr.2006.07.029

[28] Frisbee JC Brooks SD Stanley SC d’Audiffret AC. An unpredictable chronic mild stress protocol for instigating depressive symptoms, behavioral changes and negative health outcomes in rodents. *J Vis Exp.* (2015) 106:e53109. 10.3791/53109 26650668 PMC4692768

[29] Kobayashi H Gil-Guzman E Mahran AM Sharma RK Nelson DR Thomas AJ Jr. Quality control of reactive oxygen species measurement by luminol-dependent chemiluminescence assay. *J Androl.* (2001) 22:568–74. 11451353

[30] Livak KJ Schmittgen TD. Analysis of relative gene expression data using real-time quantitative Pcr and the 2^–△△Ct^ method. *Methods.* (2001) 25:402–8. 11846609 10.1006/meth.2001.1262

[31] Qi C Ding M Li S Zhou Q Li D Yu R Sex-dependent modulation of immune development in mice by secretory Iga–coated *Lactobacillus reuteri* isolated from breast milk. *J Dairy Sci.* (2021) 104:3863–75. 10.3168/jds.2020-19437 33612242

[32] Dhingra D Bansal Y. Antidepressant-like activity of beta-carotene in unstressed and chronic unpredictable mild stressed mice. *J Funct Foods.* (2014) 7:425–34.

[33] Orosco M Rouch C Beslot F Feurte S Regnault A Dauge V. Alpha-lactalbumin-enriched diets enhance serotonin release and induce anxiolytic and rewarding effects in the rat. *Behav Brain Res.* (2004) 148:1–10. 10.1016/s0166-4328(03)00153-0 14684242

[34] Seekatz AM Theriot CM Molloy CT Wozniak KL Bergin IL Young VB. Fecal microbiota transplantation eliminates clostridium difficile in a murine model of relapsing disease. *Infect Immun.* (2015) 83:3838–46. 10.1128/IAI.00459-15 26169276 PMC4567621

[35] Ding F Wu J Liu C Bian Q Qiu W-Q Ma Q Effect of xiaoyaosan on colon morphology and intestinal permeability in rats with chronic unpredictable mild stress. *Front Pharmacol.* (2020) 11:1069. 10.3389/fphar.2020.01069 32765272 PMC7378849

[36] Santos J Saunders P Hanssen N Yang PC Perdue MH. Corticotropin-releasing hormone mimics stress-induced colonic epithelial patophysiology in the rat. *Am J Physiol.* (1999) 277:G391–9. 10.1152/ajpgi.1999.277.2.G391 10444454

[37] Li S Qi C Zhu H Yu R Xie C Peng Y *Lactobacillus reuteri* improves gut barrier function and affects diurnal variation of the gut microbiota in mice fed a high-fat diet. *Food Funct.* (2019) 10:4705–15. 10.1039/c9fo00417c 31304501

[38] Duda W Curzytek K Kubera M Iciek MG Kowalczyk-Pachel D Bilska-Wilkosz A The effect of chronic mild stress and imipramine on the markers of oxidative stress and antioxidant system in rat liver. *Neurotox Res.* (2016) 30:173–84. 10.1007/s12640-016-9614-8 26961706 PMC4947122

[39] Zhu X Sun-Waterhouse D Tao Q Li W Shu D Cui C. The enhanced serotonin (5-Ht) synthesis and anti-oxidative roles of trp oligopeptide in combating anxious depression C57bl/6 Mice. *J Funct Foods.* (2020) 67:103859. 10.1016/j.jff.2020.103859

[40] Asaad GF Ahmed RF. Antidepressant activity of alpha-lactalbumin in chronic unpredictable stress model in Swiss albino mice. *Open Access Maced J Med Sci.* (2020) 8:93–9.

[41] Chang L Wei Y Hashimoto K. Brain-gut-microbiota axis in depression: a historical overview and future directions. *Brain Res Bull.* (2022) 182:44–56. 10.1016/j.brainresbull.2022.02.004 35151796

[42] Duan J Huang Y Tan X Chai T Wu J Zhang H Characterization of gut microbiome in mice model of depression with divergent response to escitalopram treatment. *Transl Psychiatry.* (2021) 11:303. 10.1038/s41398-021-01428-1 34016954 PMC8138009

[43] Blaser MJ. *Probiotic Compositions for Improving Metabolism and Immunity: U.S. Patent 10,653.* New York, NY: New York University (2020). p. 728

[44] Flint HJ Bayer EA Rincon MT Lamed R White BA. Polysaccharide utilization by gut bacteria: potential for new insights from genomic analysis. *Nat Rev Microbiol.* (2008) 6:121–31. 10.1038/nrmicro1817 18180751

[45] Devendran S Shrestha R Alves JMP Wolf PG Ly L Hernandez AG Clostridium scindens Atcc 35704: integration of nutritional requirements, the complete genome sequence, and global transcriptional responses to bile acids. *Appl Environ Microbiol.* (2019) 85:e00052–19. 10.1128/aem.00052-19 30737348 PMC6585500

[46] Zgheib R Anani H Meng MM Mailhe M Ricaboni D Morand A New human-associated species of the family atopobiaceae and proposal to reclassify members of the genus *Olsenella*. *Int J Syst Evol Microbiol.* (2021) 71:4819. 10.1099/ijsem.0.004819 34047688

[47] Björkroth K Koort J. *Lactic Acid Bacteria: Taxonomy and Biodiversity.* Amsterdam: Elsevier (2016). p. 45–8. 10.1016/B978-0-08-100596-5.00864-7

[48] Wang S Ishima T Zhang J Qu Y Chang L Pu Y Ingestion of *Lactobacillus intestinalis* and *Lactobacillus reuteri* causes depression- and anhedonia-like phenotypes in antibiotic-treated mice via the vagus nerve. *J Neuroinflammation.* (2020) 17:241. 10.1186/s12974-020-01916-z 32799901 PMC7429467

[49] Agus A Planchais J Sokol H. Gut microbiota regulation of tryptophan metabolism in health and disease. *Cell Host Microbe.* (2018) 23:716–24. 10.1016/j.chom.2018.05.003 29902437

[50] Juricek L Coumoul X. The aryl hydrocarbon receptor and the nervous system. *Int J Mol Sci.* (2018) 19:2504. 10.3390/ijms19092504 30149528 PMC6163841

[51] Ma N He T Johnston LJ Ma X. Host-microbiome interactions: the aryl hydrocarbon receptor as a critical node in tryptophan metabolites to brain signaling. *Gut Microbes.* (2020) 11:1203–19. 10.1080/19490976.2020.1758008 32401136 PMC7524279

[52] Ueno H Shimada A Suemitsu S Murakami S Kitamura N Wani K Anti-depressive-like effect of 2-phenylethanol inhalation in mice. *Biomed Pharmacother.* (2019) 111:1499–506. 10.1016/j.biopha.2018.10.073 30415864

[53] Kaddurah-Daouk R Bogdanov MB Wikoff WR Zhu H Boyle SH Churchill E Pharmacometabolomic mapping of early biochemical changes induced by sertraline and placebo. *Transl Psychiatry.* (2013) 3:e223–34. 10.1038/tp.2012.142 23340506 PMC3566722

[54] Herken H Gurel A Selek S Armutcu F Ozen ME Bulut M Adenosine deaminase, nitric oxide, superoxide dismutase, and xanthine oxidase in patients with major depression: impact of antidepressant treatment. *Arch Med Res.* (2007) 38:247–52. 10.1016/j.arcmed.2006.10.005 17227736

[55] Xu XJ Zheng P Ren GP Liu ML Mu J Guo J 2,4-dihydroxypyrimidine is a potential urinary metabolite biomarker for diagnosing bipolar disorder. *Mol Biosyst.* (2014) 10:813–9. 10.1039/c3mb70614a 24457555

[56] Micheli V Camici M Tozzi MG Ipata PL Sestini S Bertelli M Neurological disorders of purine and pyrimidine metabolism. *Curr Top Med Chem.* (2011) 11:923–47. 10.2174/156802611795347645 21401501

