# Supplementary material for: The Antidepressant Effect of Deoiled Sunflower Seeds on Chronic Unpredictable Mild Stress in Mice Through Regulation of Microbiota–Gut–Brain Axis
Source: Front Nutr. 2022 Jul 1;9:908297. doi: 10.3389/fnut.2022.908297 (PMC9289741; doi:10.3389/fnut.2022.908297)
Supplement: Supplementary file 1 [file Data_Sheet_1.docx]

***Supplementary Material***

# Supplementary methods

DNA extraction and 16S rRNA gene sequencing

Lysis buffer (1 mL) was added to the suspension of SIgA-targeted bacteria or precipitates of fecal fermentation samples. The suspension was then transferred to a cracking tube containing 0.3 g of 0.15 mm and 0.1 g of 0.7 mm zirconia/silicon beads (Shanghai Shenggong Biological Engineering Co. Ltd, China). Samples were homogenized using a Multifunctional Tissue Homogenizer (Bertin, France) for two cycles of bead-beating at 6,300g for 35 s and cooled for 45 s on ice. After centrifugation (15,000g, 4 °C, 5 min), the supernatant was transferred to a 2 mL Eppendorf (EP) tube and 300 μL lysis buffer was added to the precipitate. The above steps were repeated to incorporate the supernatant into a 2 mL EP tube (MACKLIN, China). The supernatant was mixed with 300 μL of ammonium acetate and then centrifuged for 10 min (15,000g, 4 °C). The DNA was then precipitated by adding 1 volume of isopropanol to the aqueous phase. Precipitated DNA was pelleted (15 min, 15,000g, 4 °C), washed with 1 mL 75% EtOH (5 min, 15,000g, 4 °C), dried (15 min, no heat, autorun setting), and resuspended in 100 μL TE buffer ( pH 7; 50 °C for 30 min). The DNA was then treated with 10 mg mL^−1^ RNase A (Shanghai Shenggong Biological Engineering Co. Ltd, China) before purification (Ezup column bacterial genomic DNA extraction kit, Shanghai Shenggong Biological Engineering Co.Ltd, China) and elution in 50 μL CE buffer for further ana-lysis^1^. DNA concentrations were determined using a Qubit3.0 Fluorometer (Invitrogen, Carlsbad, CA, USA).

Microbial profiles were analyzed by 16S rDNA sequencing at BGI Co., Ltd (Wuhan, China). To maximize the effective length of the MiSeq 250PE and 300PE sequencing reads, a region of approximately 469 bp encompassing the V3 and V4 hypervariable regions of the 16S rRNA gene was targeted for sequencing. The PCR primers used to amplify V3, and V4 hypervariable regions were as follows: forward 5'- CCT ACG GRR BGC ASC AGK VRV GAA T -3' and reverse 5'- GGA CTA CNV GGG TWT CTA ATC C -3'. In addition, an indexed linker was added to the end of the 16S rDNA PCR product for next-generation sequencing (Illumina, San Diego, CA, USA). First-round PCR products were used as templates for the second round of PCR amplicon enrichment (94 °C for 3 min, followed by 24 cycles at 94 °C for 5 s, 57 °C for 90 s, and 72 °C for 10 s, and a final extension at 72 °C for 5 min). PCR reactions were performed in triplicate using a 25 μL mixture containing 2.5 μL of TransStart Buffer, 2 μL of dNTPs, 1 μL of each primer, and 20 ng of template DNA. DNA library concentration was validated using a Qubit 3.0 Fluorometer. According to the manufacturer's instructions, the libraries were quantified to 10 nM and subsequently multiplexed and loaded on an Illumina MiSeq instrument. The libraries were quantified to 10 nM and subsequently multiplexed and loaded on an Illumina MiSeq instrument (Illumina, San Diego, CA, USA). Sequencing was performed using PE250/300 paired-end; image analysis and base calling were performed using the MiSeq Control Software embedded in the MiSeq instrument.

The raw data were filtered to eliminate the adapter pollution and low quality to obtain clean reads, then paired-end reads with overlap were merged to tags using FLASH (Fast Length Adjustment of Short reads, v1.2.11)^2^. The tags were clustered to OTU with a 97% threshold using USEARCH (v7.0.1090)^3^. All tags were mapped to each OTU representative sequence using USEARCH GLOBAL. OTU representative sequences were taxonomically classified using a ribosomal database project (RDP) classifier (v.2.2)^4^ on the database Greengene, using the 0.6 confidence value as a cut-off.

**Reference**

1 Z. Yu and M. Morrison, *Biotechniques*, 2004, **36**, 808–812.

2 T. Magoč and S. L. Salzberg, *Bioinformatics*, 2011, **27**, 2957–2963.

3 R. C. Edgar, *Nat. Methods*, 2013, **10**, 996–998.

4 Q. Wang, G. M. Garrity, J. M. Tiedje and J. R. Cole, *Appl. Environ. Microbiol.*, 2007, **73**, 5261–5267.

1. **Supplementary Figures and Tables**

## Supplementary Figures


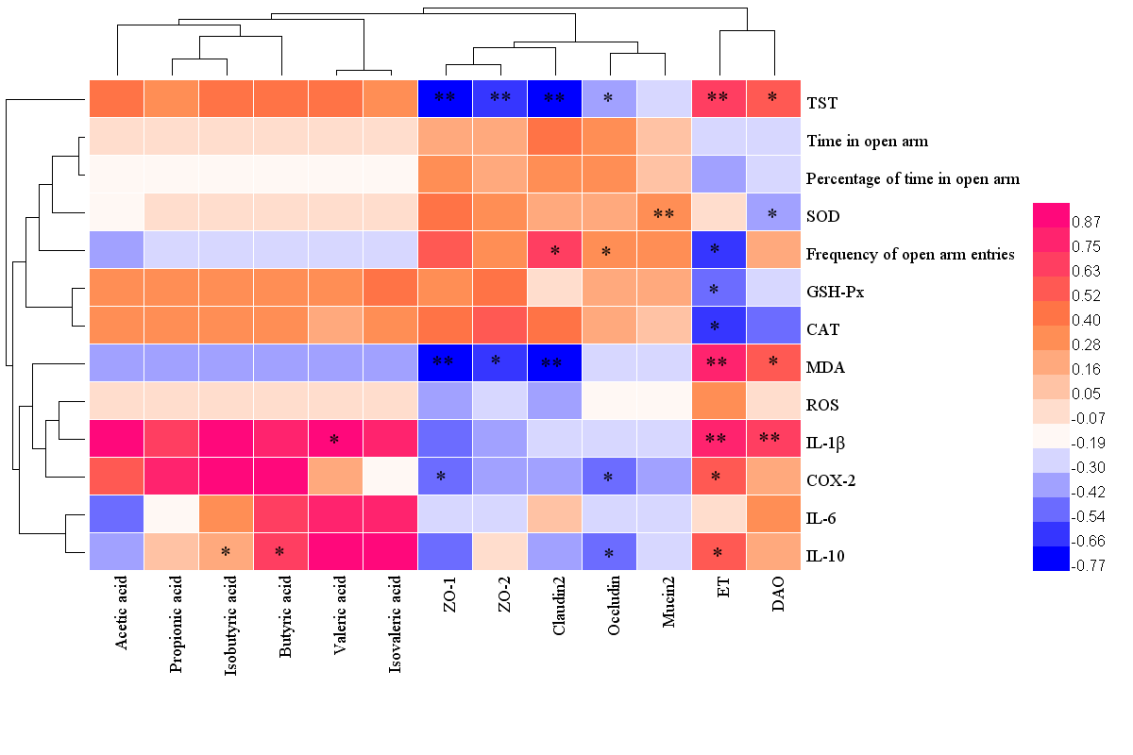


**Supplementary Figure 1** Correlation heat map of SCFA, intestinal mucosal barrier, and biochemical indicators

Note: The x-axis and y-axis of the heat map are SCFA, intestinal mucosal barrier, and biochemical respectively, and the *R* value and *P* value are obtained by calculation. The *R* value is represented by different colors in figure, and the color card on the right is the color interval of different *R* values. * was significantly correlated at the 0.05 level; ** was significantly correlated at the 0.01 level.

## Supplementary Tables

**Supplementary Tables S1**. Amino acid composition of protein in the diet

| Amino acid (g/100g protein) | CON/MOD  Soy protein | DSFS | WPC |
| --- | --- | --- | --- |
| Lysine | 6.40 | 3.35 | 10.25 |
| Methionine + Cystine | 2.58 | 3.18 | 4.18 |
| Threonine | 4.10 | 5.40 | 7.82 |
| Isoleucine | 5.30 | 5.08 | 8.25 |
| Leucine | 8.06 | 6.73 | 12.03 |
| Phenylalanine + Tyrosine | 8.61 | 14.15 | 6.65 |
| Valine | 4.93 | 6.11 | 7.54 |
| Histidine | 2.77 | 4.94 | 2.14 |
| tryptophan | 1.06 | 2.35 | 1.22 |
| Glutamate | 17.89 | 45.06 | 22.92 |
| Arginine | 8.12 | 16.58 | 3.06 |
| aspartic acid | 11.43 | 19.97 | 13.23 |
| Glycine | 4.57 | 9.80 | 2.25 |
| Proline | 5.33 | 8.12 | 7.86 |
| Alanine | 4.41 | 7.96 | 6.26 |
| Serine | 5.28 | 6.07 | 4.77 |

DSFS, deoiled and dechlorogenic acid sunflower seeds; WPC, whey protein..

**Supplementary Tables S2** Composition of animal feed (g/kg)

| Composition | CON | MOD | DSFS | WPC |
| --- | --- | --- | --- | --- |
| Protein  (Protein material) | 140 (175) | 140 (175) | 140 (367) | 140  (211) |
| Sucrose | 100.3 | 100.3 | 100.3 | 100.3 |
| Corn starch | 548 | 548 | 460.8 | 534.4 |
| Sunflower oil | 85 | 85 | 0 | 82.2 |
| Mixed minerals (AIN-93M-MX) | 27.3 | 27.3 | 27.3 | 27.3 |
| Mixed vitamins | 10 | 10 | 10 | 10 |
| Fiber | 50 | 50 | 32.1 | 47.7 |
| Choline | 2.3 | 2.3 | 2.3 | 2.3 |
| Total protein supply (%) | 14.7 | 14.7 | 14.7 | 14.7 |
| Total oil and fat supply (%) | 18.1 | 18.1 | 18.1 | 18.1 |
| Total carbohydrate supply (%) | 75.8 | 75.8 | 75.8 | 75.8 |
| Energy supply ratio（KJ/g） | 21.39 | 21.39 | 21.39 | 21.39 |

Note: The mineral mixture was prepared according to AIN-93M-MX; Mixed vitamins (mg/kg): VA 4000 IU; VD_3_ 1000 IU; VE 50 IU; VB_3_ 30; VB_5_ 16; VB_6_ 7; VB_1_ 6; VB_2_ 6; VB_11_ 2; VK_3_ 2; VB_12_ 0.01; VH 0.2 mg/kg. Protein: actual protein content per kg of diet; Protein material: actual dose of protein material added per kg of diet.

**Supplementary Tables** **S3** Primer sequences of the genes

| Gene | Forward primer (5’->3’) | Reverse primer R (5’->3’) |
| --- | --- | --- |
| β-actin | GGCTGTATTCCCCTCCATCG | CCAGTTGGTAACAATGCCATGT |
| IL-1β | GCAACTGTTCCTGAACTCAACT | ATCTTTTGGGGTCCGTCAACT |
| IL-6 | TGGAGTCACAGAAGGAGTGGCTAAG | TCTGACCACAGTGAGGAATGTCCAC |
| IL-10 | TGCCAAGCCTTATCGGAAAT | TTTTCCAAGGAGTTGTTTCCGTTA |
| Cox-2 | GAAGTCTTTGGTCTGGTGCCT | GCTCCTGCTTG AGTATGTCG |
| ZO-1 | TACCTCTTGAGCCTTGAACTT | CGTGCTGATGTGCCATAATA |
| ZO-2 | GCCAAAACCCAGAACAAAGA | ACTGCTCTCTCCCACCTCCT |
| Occludin | GTGTGGTTGATCCCCAGGAG | TCGCTTGCCATGATGGTGA |
| Claudin-2 | CCCAGGCCATGATGGTGA | TCATGCCCACCACAGAGATAAT |
| Mucin-2 | CCCAGAAGGGACTGTGTATG | TGCAGACACACTGCTCACA |

Abbreviations: IL-1β, interleukin 1β; IL-6, interleukin 6; IL-10, interleukin 10; Cox-2, cyclooxygenase-2; ZO-1, zonula occludens 1; ZO-2, zonula occludens 2.
